# An Assessment of Malaria Parasite Density among HIV/AIDS-Subjects at Different Levels of CD4 T-Cells Prior to Antimalarial Therapy at Chulaimbo Sub-County Hospital, Western Kenya

**DOI:** 10.1155/2019/5697383

**Published:** 2019-07-01

**Authors:** J. K. Kirinyet

**Affiliations:** Department of Medical Microbiology & Parasitology, School of Medicine, College of Health Sciences, Moi University, P.O. Box 4606-30100, Eldoret, Kenya

## Abstract

**Background:**

Malaria and HIV/AIDS infections are among the major public health concerns in sub-Saharan Africa, where they are associated with high morbidity and mortality. Recent findings indicate that individual people living with HIV/AIDS (PLWHA) with lower levels of CD4 T-cell count below 200/mm^3^ tend to experience higher mean malaria parasite densities than their counterparts with higher CD4 T-cells counts.

**Aim:**

The study was conducted to assess the pattern of malaria parasite density at different levels of CD4 T-cells among people living with HIV/AIDS in Western part of Kenya.

**Subjects and Methods:**

A randomized antimalarial treatment study among 126 people living with HIV/AIDS was conducted at Chulaimbo Sub-County Hospital, Western Kenya. All the participants enrolled into the study had their blood samples assessed for malaria parasite densities before commencement of antimalarial therapy and the results correlated with their CD4 T-cells levels obtained from their respective files.

**Results:**

Mean malaria parasite density on pretreatment samples was 43,168 parasites /*μ*L of blood, median was 17,720, and mode was 4,000. Male participants had a higher geometrical mean parasite density (26,424) compared to females' (15,346) (p = 0.03). Low CD4 counts were associated with high density malaria parasitaemia and consequently, very high CD4 counts seemed to exhibit low malaria parasite density among PLWHA. An insignificant negative correlation, however, between CD4 T-cells count and malaria parasite densities was noted (p = 0.169).

**Conclusion:**

The study was able to establish higher parasite density among individuals with ≤200 cells/*μ*L than their counterparts with >200 cells/*μ*L of CD4 T-cell levels in PLWHA resident in Western Kenya. Secondly, males significantly had a higher geometrical mean parasite density than females regardless of their CD4 status. It is anticipated that the results from this study could be used/applied in developing interventional measures to address malaria/HIV-AIDS coinfections aimed at saving life, particularly in the sub-Saharan African region where the two infections are rampant.

## 1. Introduction

In sub-Saharan African countries, HIV/AIDS and malaria coinfection are among the most important public health burden/concern evidently contributing to high morbidity and mortality in the region. Over the years, the prevalence, morbidity, and mortality associated with the two coinfections have been changing globally and the trend has been varying from one region to another. Globally, it was estimated that 212 million cases of malaria occurred in 2015, representing a 22% decline from the figures reported in the year 2000 and a further decline of 14% since 2010. The African and Asian regions/continents where 90% of world malaria cases were reported during the same period had experienced minimal disease decline by between 2 adn 7% [1].

In sub-Saharan Africa, malaria cases decreased from 146 million in 2005 to 114 million in 2015. However, high infection rates in children aged 2–10 have been widely reported, though mortality rates declined to 29% [1]. In Kenya, it is estimated that malaria episodes accounted for 6.7 million new clinical malaria cases and 4,000 deaths in 2015, especially among communities living in Western Kenya [2].

The pandemic of HIV/AIDS infection has persistently remained a major health concern since its discovery in the 1980s. Globally, people living with HIV/AIDS as of December 2015 were estimated at 36.7 million (all ages) with new infection (all ages) of 2.1 million. This represents an increase of 6.4% from 33 million in 2007 estimates [3] with 19 million from Eastern and South African regions. New infections reported in the two regions at the time were 960, 000 with about 1.1 million deaths. Kenya as a country ranks fourth-largest in HIV/AIDS epidemic in the world alongside Mozambique and Uganda with approximately 1.6 million people living with HIV by end of 2016 and an estimated 36,000 deaths due to AIDS-related illnesses [4]. Further, western part of the country is the most affected region in the country with highest rates of infection between 19.3% and 25.7% [5].

Malaria and HIV/AIDS overlapping geographical distribution has raised a lot of concerns and challenges in public health, with HIV infection increasing malaria susceptibility and reducing the efficacy of antimalarial drugs, while malaria infection has been found to increase the risk of HIV disease progression and mother-to-child transmission of HIV [6].

In endemic malaria areas of sub-Saharan Africa, HIV has been found to increase the risk of malaria infection especially in subjects with increased immunosuppression, while in unstable malaria areas, HIV-infected ones are at high risk of developing complicated and severe malaria that may result in death [7].

Malaria and HIV/AIDS coinfections are increasingly reported worldwide [8] and the interaction may lead to inadequately/poorly understood effect(s) on the disease(s) outcome and clinical presentation. In HIV/malaria coinfected patients, a significant variation in parasite density has been reported [9]. Interaction between malaria and HIV/AIDS infections and to some extent TB in areas where the three infections overlap has been reported to increase malaria parasitaemia levels, acid fast bacilli (AFB) count, and decreased Hgb levels [10]. In a study carried out among adults in Gabon, Central Africa, low CD4 T-cells counts (<200 cells/*μ*L) in HIV positive subjects were associated with high parasite density levels [11]. Several studies on the association of the two infections in Western Kenya have been conducted in the past but no similar study has been done for the patients presenting at the Chulaimbo Sub-County Hospital under antimalarial directly observed therapy (DOT). The study was conducted to assess the pattern of malaria parasitaemia densities in different levels of CD4 T-cells among people living with HIV/AIDS in the surrounding areas within 10 km radius of the site. The findings will enable formulation of targeted interventions to address the coinfections in the region.

## 2. Materials and Methods

### 2.1. Study Area and Site

**Figure 1:**
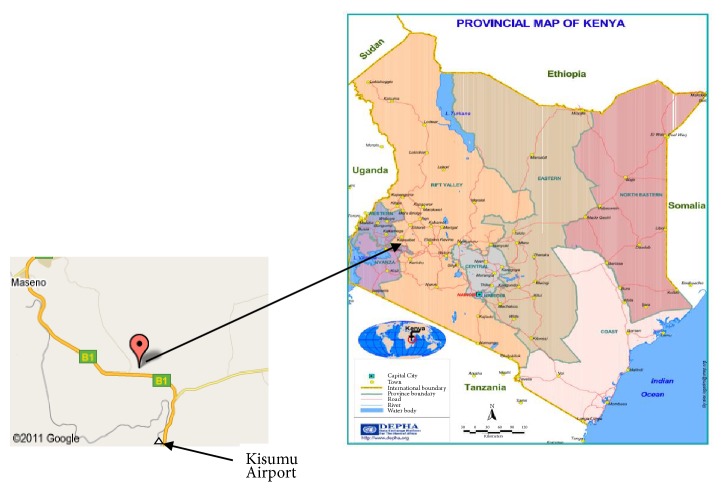
Study area in Kisumu, Kenya. Source: The Data Platform for the Horn of Africa (DEPHA) (www.depha.org) [12].

The study was conducted at Chulaimbo Health Centre, located some 24.1km north of Kisumu City in Western part of Kenya (Figure 1). The study area is located at latitude: 0°06′07′′S and longitude: 34°45′42′′E with an elevation of 1174 m above sea level. It receives mean annual rainfall of 1800 mm with a mean annual temperature of 25-35°C.

### 2.2. Study Design and Setting

A prospective study was conducted at a health facility between November 2014 and October 2015 among people living with HIV/AIDS (PLWHA) in a malaria endemic zone within 10 km radius. Western Kenya, particularly the former Nyanza Province, is considered both malaria and HIV/AIDS endemic area [2, 5]. According to Asito* et al*. (2011) [13], residents of Kisumu county (a malaria holoendemic region) receive between 100 and 300 infective mosquito bites per annum.

### 2.3. Patients' Eligibility and Enrollment

A total of 13,057 suspected malaria patients were screened at the facility and 5,867 had malaria. Of the positive malaria cases, 126 individuals with HIV/AIDS coinfection, aged between 2 and 72 years, met the inclusion criteria to participate in the study. They comprised both male and female subjects drawn from among those attending HIV management care within the health facility. Eligibility to the study was based on malaria positive slide with monoinfection of* P. Falciparum; a*sexual parasitaemia of ≥1000 and ≤200,000 parasites/*μ*L; febrile state with an auxiliary temperature of ≥37.5°C; haemoglobin (Hb) of ≥5 g/dL of blood and willing to give consent. Ineligibility to the study was based on multiple infections with* Plasmodium* species; aged below 2 years; severe anaemia (Hb ≤5.0 g/dL); females with a positive pregnancy test; history of taking antimalarial drugs within 48 hours prior to visiting the health facility; severe opportunistic infections (OI) and recipient of second-line regimen of ARV treatment (Kaletra).

### 2.4. Sample Size Determination

The sample size required for this study was calculated based on 95% and 90% confidence levels and margin of error of 5% and 10%, respectively, for the two drugs in accordance with Lemeshow* et al*. (1990) formula. The minimum sample size (n) was thus calculated as

(1) $$\begin{array}{c} n=\frac{2{\left(Z_{1-\propto /2}+Z_{1-\beta }\right)}^{2}\delta^{2}}{{\left(M1–M_{2}\right)}^{2}} \end{array}$$

where n is the minimum sample size, Z_1−∝/2_ is the standard error of first drug (Quinine) = 1.96(95%), Z_1-*β*_ is standard error of second drug (Coartem®) = 1.282 (90%), *δ* is standard deviation of Quinine (=12), [14], M_1_ is mean parasite clearance of Quinine (41 hr), M_2_ is mean parasite clearance of Coartem® (36 hr), and n = 2(1.96 +1.282)^2^ × 12^2^/(41-36)^2^ = 121 patients.

### 2.5. Sample Collection and Processing Procedures

Blood smears (both thick and thin) were collected from each participant on day zero (D0) prior to commencement of treatment. Thin blood smears were fixed using absolute methanol prestaining and both slides were stained with 10% buffered Giemsa stain (pH 7.2) for 10 minutes. Thin blood smears were examined for speciation while thick ones were quantitatively examined for parasitaemia from which parasite density was calculated. Additional blood sample (0.02 ml) was taken from the same prick for the estimation of haemoglobin (Hb) using HemoCue AB, Angelhom, Sweden equipment.

Malaria parasites were counted in all positive thick blood smears and parasitaemia quantified per 200 white blood cells (WBC) counted in all fields. Ultimate parasitaemia value was calculated and given per microlitre (*μ*L) of blood using McKenzie* et al*. (2005) [15] mathematical formula:

(2) Malaria  parasites  per  μL  blood=No.  malaria  parasites  counted  x  8 000200  WBC

An average of 8000 leucocytes per *μ*L blood (range 4000–11000 WBC/*μ*L blood) was used as the standard for the study population.

Routine CD4 cells count was conducted among all the PLWHA attending the facility as part of management care and their data were available in individual files kept at the clinic.

### 2.6. Data Management and Analysis

Raw data was stored in Microsoft Excel Spreadsheet program and back-ups in flash discs and hand written copy in analysis notebook. After validation, data analysis was done using computer software SPSS (Statistical Package for Social Scientists) version 12 to generate means, standard deviation, median, and frequency distributions. To test for significant difference between groups, t-test was used for continuous variables such as age and parasitaemia that were normally distributed.

### 2.7. Ethical Approval

This study was granted approval by Institutional Research & Ethical Committee (IREC) of Moi Teaching & Referral Hospital /Moi University, School of Medicine, Eldoret–No: FAN: IREC 000421 and the Scientific Steering Committee (SSC)/National Ethical Research Committee (NERC), KEMRI, Nairobi–No: SSC No 1495. All confidentiality regarding the study was professionally observed throughout the period of data collection. Access to participants' data at the study site was prohibited to persons other than the Principal Investigator. All participants to the study were assigned a study number at the point of recruitment to conceal their identity.

### 2.8. Quality Assurance

Both good laboratory and clinical practices were observed at study site by professionally qualified personnel through continuous monitoring by the Safety Monitor, Principal Investigator (PI), and the clinical consultant. Giemsa stain and chemicals such as methanol and buffer salts were sourced from Sigma-Aldrich (Catalogue No: 65637-25G; M1775-1GA; P3288-12VL, respectively). Training and quality assurance in microscopy for study site technologists was done continuously by the PI. All subjects' slides were taken to Malaria Unit, Centre for Biotechnological Research and Development, KEMRI, Nairobi, for reexamination and validation. Random microscopy checks of malaria slides were regularly done at the site by the PI for quality control.

## 3. Results

### 3.1. Demographic and Clinical Characteristics

A total of 126 participants were recruited into the study, 83.3% (105/126) of which were adults while children participants were 16.7% (21/126). Mean age of the participants was 31.3 and median was 31, the most frequent age was 30 years, and standard deviation was 14.7 in a range of between 2 and 72 years (Table 1).

The lowest recorded parasite density pretreatment among the participants was 1,000 parasites/*μ*L of blood while the highest recorded density was 199,000 parasites /*μ*L of blood. The mean parasite density pretreatment was 43,168 parasites /*μ*L of blood and the median was 17,720 while the mode was 4,000. The mean temperature on recruitment, prior to commencement of antimalarial therapy, was 37.6°C (slightly febrile), and median temperature was 37.4°C (normal), while the mode was 37.0°C. Temperature range was 34.8°C-40.9°C. The mean blood haemoglobin of participants was 11.7g/dl while median and mode were 11.9g/dl and 12.5g/dl, respectively. Mean CD4 cells count was 519.3 with a median of 478 and the mode was 449 with a range of between 12 and 1567.

### 3.2. Comparison of Geometrical Means of Parasite Density, Temperature, Hb, and Temperature within Gender Day 0

The mean age of the participants was 32.1 and 30.2 for females and males, respectively (p > 0.524) (Table 2). Data revealed a higher geometrical mean parasite density for males (26,424) than for females (15,346) with a significant difference within gender (p < 0.03). The mean haemoglobin for males was 12.4, significantly different from that of females of 11.2 (p > 0.04).

### 3.3. Mean Malaria Parasite Density in Different Age Groups Pretreatment at Day 0

**Figure 2:**
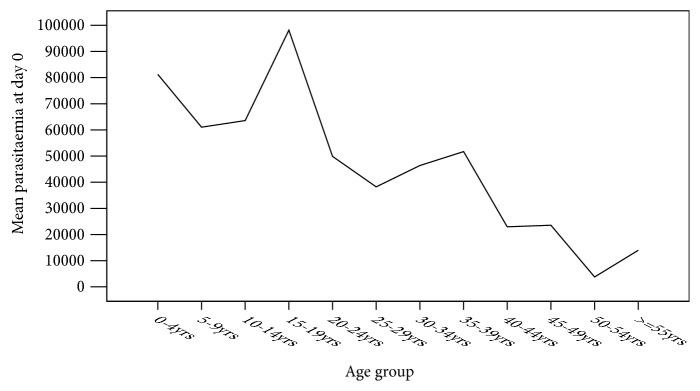
Mean parasite density in different age groups on day 0.

The ages below 5 years of age and those between 10 and 19 years had higher mean parasite density compared to the other age groups (Figure 2).

### 3.4. Malaria Parasite Density Relative to CD4 Cells Count Prior to Antimalarial Therapy

**Figure 3:**
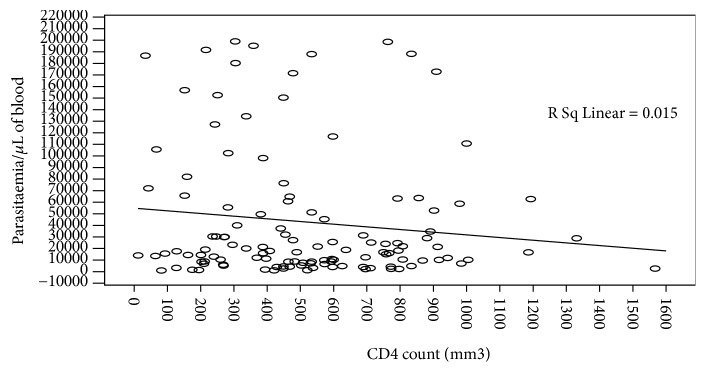
Density of malaria parasites relative to CD4 T-cells count* on day 0*.

**Figure 4:**
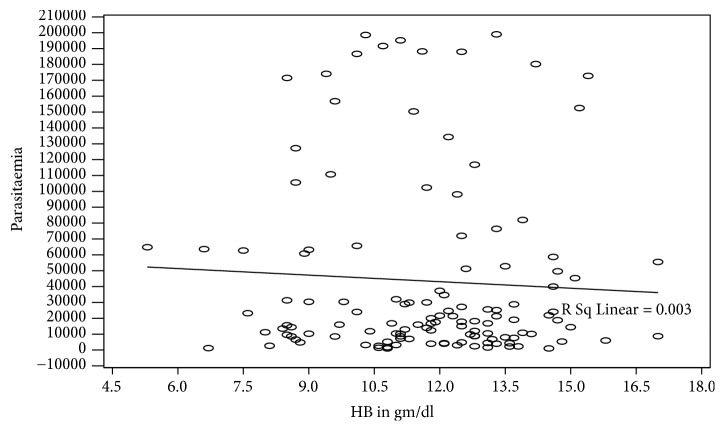
Haemoglobin and parasite density prior to antimalarial treatment.

Participating individuals' CD4 values were analyzed against parasitaemia prior to commencement of antimalarial therapy on day 0 (Figure 3, Table 3, and Figure 4) and results showed a negative correlation between CD4 count and malaria parasite densities, but the correlation, however, was statistically insignificant (p > 0.169). Data presented (Figure 3) seem to suggest that low CD4 counts was associated with high density malaria parasitaemia. Consequently, very high CD4 counts seemed to exhibit low malaria parasite density among people living with HIV/AIDS. The geometrical mean parasitaemia (Gm) in the four categories of CD4 count (Table 3) indicates that individuals with <200 CD4 cells/mm^3^ had a higher SD and Std error than their counterparts whose CD4 counts were above 200/mm^3^. When Hb versus parasitaemia data were analyzed and correlated, the correlation, however, was not statistically significant, p=0.543.

## 4. Discussion

It is apparent that association between the two infections (malaria and HIV) at the biological, immunological, and drug levels does exist. A significant variation in parasite density has further been observed among malaria/HIV coinfected groups [9]. Other studies indicate that HIV-1 positive individuals tend to have a significantly higher mean malaria parasite density than their HIV-1 negative counterparts [16]. Triple infections of malaria, HIV/AIDS, and TB have been reported to increase malaria parasite densities, acid fast bacilli (AFB) count, and decreased haemoglobin levels among individuals in endemic areas [10]. These coinfections may worsen the immune response to either of the diseases [9]; cause transient increases in HIV-1 viral load that may progress to AIDS disease [17, 18]; and also predict an increased rate of AIDS-related disease (ARD) among PLWHA.

The current study findings showed that individuals living with HIV within age brackets below 5 years and those between 10 and 19 years had the highest malaria density of about 95,000/ *μ*L regardless of their CD4 T-cells status. The current results seem to resonate well with data obtained from General Hospital; Makarfi Kaduna, Nigeria, where children aged 5–15 years had a statistically significant higher parasitaemia density compared to other age groups [19]. And in Cameroon, West Africa, a geometric mean malaria parasite density (GMPD) among children aged 1–15 years was significantly higher in those presented with fever and moderate anaemia than their respective counterparts [20]. The higher malaria parasitaemia densities among children aged below 5 years and individuals aged between 10 and 19 years old in this study could be associated with immunological factors. Malaria infection has been associated with high viral load while HIV reputed to cause more clinical malaria. These factors, together with generally low immunity in children under 5 years, could explain the high parasitaemia levels witnessed in the 0-5 age group [21], which could have been the case in the current study. But in the Cameroon study, however, a significantly higher geometric mean parasite density among children whose CD4 T- cells ≥500 cells/*μ*L (491.3, P = 0.003) than the values below was observed [20]. In areas of stable malaria, adolescents (10-19 year age group) usually have higher risks of malaria parasitaemia because of immunological as well as hormonal factors that come into play during their development [22]. And among Gabonese women living with HIV, a higher median malaria parasitaemia was reported in individuals with a CD4 T-cell count below 200/*μ*L (p=0.03) [11]. There is a possibility that the observed differences in parasite density may be encountered due to geographical differences in study population and levels of malaria endemicity.

The added immune deficiency syndrome due to HIV depletion of CD4 T-cells may explain the high parasite density among people living with HIV/AIDS as indicated in the current study. The infection of HIV/AIDS has been known not only to induce depletion of CD4 T-cells but also to reduce CD8 T-cells causing downmodulation, reduction in T-cell subpopulation, and defective cell mediated immunity against any microbial infection [23, 24].

Further, this study also documented a general higher geometrical mean malaria parasite density in males than in females with a significant difference within gender (p = 0.03), which was comparable with data obtained from Kaduna State, Nigeria [19].

## 5. Conclusions

This study established a higher parasite density among individual PLWHA with CD4 T-cells below 200/*μ*L than their counterparts with cells above 200/*μ*L) levels. Further, males significantly had a higher geometrical mean parasite density than females regardless of their CD4 status. It is anticipated that the results from this study could be used/applied in developing interventional measures to address malaria/HIV-AIDS coinfections aimed at saving life, particularly in the sub-Saharan African region where the two infections are rampant.

## Figures and Tables

**Table 1:** Profile of parasite density, temperature, Hb, and CD4 cells count of participants.

Parameter	Mean	Median	Mode	SD	Range
Age	31.3	31	30	14.7	2-72
Haemoglobin in g/dL	11.7	11.9	12.5	2.25	5.3-17
Parasitaemia density per *μ*L	43168	17720	4,000	5602	1000-199000
Temp. in degrees Celsius	37.6	37.4	37.0	1.34	34.8-40.9
CD4 per *μ*L	519.3	478	449	296.3	12-1567

Hb: haemoglobin; SD: standard deviation; g/dl: grammes per decilitre; *μ*L: microlitre.

**Table 2:** Geometrical means of age, parasite density, temperature, and Hb between gender day 0.

Parameter	Male n-48	Female n-78	p-value
Mean age	30.2	32.1	0.524 NS
Gm parasitaemia	26,424	15,346	0.03 S
Mean Hb	12.4	11.2	0.04 S
Mean temperature in °C	37.7	37.5	0.447 NS

Gm: geometrical mean; Hb: haemoglobin; NS: not significant; n: number of participants.

**Table 3:** Geometric mean parasite density in the four categories of CD4 count.

CD4 Category (/mm^3^)	N	Gm Parasite density	S.D.	Std. Error	95% CI for mean		Min	Max
					Lower Bound	Upper Bound		
<200	15	4.254	.7622	.1968	3.832	4.676	3.0	5.3
200-349	24	4.499	.5192	.1060	4.279	4.718	3.7	5.3
350-499	24	4.332	.5829	.1190	4.085	4.578	3.2	5.3
>500	58	4.196	.5372	.0705	4.055	4.337	3.1	5.3
Total	121	4.290	.5789	.0526	4.186	4.394	3.0	5.3

## Data Availability

The data used to support the findings of this study are available from the corresponding author upon request.
